# Effect of Different Pretreatment of Sugar Cane Bagasse on Cellulase and Xylanases Production by the Mutant *Penicillium echinulatum* 9A02S1 Grown in Submerged Culture

**DOI:** 10.1155/2014/720740

**Published:** 2014-05-20

**Authors:** Marli Camassola, Aldo J. P. Dillon

**Affiliations:** Institute of Biotechnology, University of Caxias do Sul, P.O. Box 1352, 95070-560 Caxias do Sul, RS, Brazil

## Abstract

The main limitation to the industrial scale hydrolysis of cellulose is the cost of cellulase production. This study evaluated cellulase and xylanase enzyme production by the cellulolytic mutant *Penicillium echinulatum* 9A02S1 using pretreated sugar cane bagasse as a carbon source. Most cultures grown with pretreated bagasse showed similar enzymatic activities to or higher enzymatic activities than cultures grown with cellulose or untreated sugar cane bagasse. Higher filter paper activity (1.253 ± 0.147 U*·*mL^−1^) was detected in the medium on the sixth day of cultivation when bagasse samples were pretreated with sodium hydroxide, hydrogen peroxide, and anthraquinone. Endoglucanase enzyme production was also enhanced by pretreatment of the bagasse. Nine cultures grown with bagasse possessed higher **β**-glucosidase activities on the sixth day than the culture grown with cellulose. The highest xylanase activity was observed in cultures with cellulose and with untreated sugar cane bagasse. These results indicate that pretreated sugar cane bagasse may be able to serve as a partial or total replacement for cellulose in submerged fermentation for cellulase production using *P. echinulatum,* which could potentially reduce future production costs of enzymatic complexes capable of hydrolyzing lignocellulosic residues to form fermented syrups.

## 1. Introduction

Lignocellulosic biomass is the most abundant organic material on earth and could provide a suitable low-cost feedstock for the production of ethanol fuel and other chemicals in the future [1]. These materials generally consist of up to 75% of cellulose and hemicellulose, which are compounds that cannot be easily converted to simple monomeric sugars due to their recalcitrant nature [2]. Sugar cane bagasse is a waste product of the sugar extraction process. Currently, the sugar cane bagasse generated in the plant is consumed for energy production through cogeneration, becoming self-sustainable energy plant, and in some cases spares energy to electricity sales. This is an abundant, low-cost lignocellulosic material that could be a promising feedstock for the use as a carbon source in the fermentation medium for the production of cellulase enzymes [3]. Additionally, it contains xylan, an inducer of xylanase production [4].

Xylanases are produced on an industrial scale for use as a food additive for poultry to increase feed efficiency and in wheat flour to improve dough handling and the quality of baked products. The interest in xylanases has markedly increased recently due to the other potential industrial uses, particularly in pulping and bleaching processes, using cellulase-free preparations [5]. Cellulolytic enzymes are used in a large number of processes including supplementation of animal feeds, extraction of fruit and vegetable juices, pulp and paper manufacturing, starch processing, textile processing, and ethanol production [6–10]. Furthermore, growing concerns regarding the potential consequences of a worldwide shortage of fossil fuels, the emission of greenhouse gases, and air pollution caused by incomplete combustion of fossil fuels have resulted in an increased focus on the production of bioethanol from lignocellulosics materials [11, 12] and use of cellulases and hemicellulases in the enzymatic hydrolysis of lignocellulosic material [3].

The main limitation to the industrial scale hydrolysis of cellulose is the cost of cellulase production [13]. The challenges are to obtain cellulase-producing mutant microorganisms [14, 15] and economic cellulase production processes in combination with inexpensive sources of inducers along with total or partial recycling of enzymes.

This study evaluated cellulase and xylanase enzyme production by the cellulolytic mutant* Penicillium echinulatum* strain 9A02S1 [15] using sugar cane bagasse pretreated with different chemicals as an inducer of these enzymes. Mutants of* P. echinulatum *are potential producers of cellulases for cellulose hydrolysis because they offer relatively good thermal stability of filter paper activity (FPA) and *β*-glucosidase enzymes at 50°C, maintaining the activity for more than 80 hours [16], high levels of enzymatic production [17], and good proportions of FPA and *β*-glucosidase for the efficient hydrolysis of cellulose as compared to the cellulases of* T. reesei* [18]. Therefore, this strain has potential value for the enzymatic hydrolysis of cellulose and lignocelluloses to produce glucose syrup.

## 2. Materials and Methods 

### 2.1. Strain and Culture Media

The cellulolytic mutant* P. echinulatum* strain 9A02S1 (DMS 18942) was used in this study. The strain was obtained by exposing wild-type* P. echinulatum* strain 2HH to ultraviolet (UV) light and hydrogen peroxide (H_2_O_2_) [15]. These strains are stored in the culture collection of the Division of Process Biotechnology, Institute of Biotechnology, Caxias do Sul, Rio Grande do Sul, Brazil.

The media used were based on 10x concentrated Mandels and Reese solution (MS) [19] containing (in g·L^−1^) KH_2_PO_4_, 20; (NH_4_)_2_SO_4_, 13; CO(NH_2_)_2_, 3; MgSO_4_·7H_2_0, 3; CaCl_2_, 3; FeSO_4_·7H_2_O, 0.050; MnSO_4_·H_2_O, 0.0156; ZnSO_4_·7H_2_O, 0.014; and CoCl_2_, 0.0020. It was used swollen cellulose to prepare the strain maintaining medium. Swollen cellulose gel was produced by placing 5 g of Cellufloc 200 cellulose (Celulose e Amido Ltda, Suzano, São Paulo, Brazil), 60 mL of distilled water, and 20 glass beads (*ϕ* = 3 mm) in a 500 mL Erlenmeyer flask, which was then sealed and autoclaved at 120°C for 30 min, after which the flask and its contents were shaken for 48 h at 180 rpm, and 140 mL of distilled water was added. The swollen cellulose was stored at 4°C until use.

The strain 9A02S1 was grown and maintained on cellulose agar (C-agar) consisting of distilled water containing 1% (v/v) swollen cellulose, 10% (v/v) 10x MS, 0.1% (w/v) proteose peptone (Oxoid L85), and 2% (w/v) agar and the pH was adjusted to 6.0. Cultures were grown on C-agar slants for up to 7 days at 28°C until conidia formed and then stored at 4°C until use.

### 2.2. Obtention of Delignified Bagasse Samples

The delignification of bagasse was generally carried out using different quantities of sodium hydroxide, hydrogen peroxide, anthraquinone (AQ), and ethylenediaminetetraacetic acid (EDTA) solutions at 120°C for 20 minutes. Liquor ratios and reagent concentrations for the mixtures are shown in Table 1. The samples were ground into fragments of size between 0.5 and 1 cm.

#### 2.2.1. Enzyme Production

Shake-flask experiments were carried out in 500 mL Erlenmeyer flasks with 100 mL of production medium containing 10% (v/v) 10x MS, 1% (w/v) microcrystalline cellulose-200 or pretreated sugar cane bagasse samples from different alkaline pretreatments (Table 1), 0.2% (w/v) soy meal, and 0.1% (v/v) Tween 80. The flasks were inoculated with sufficient conidial suspension to give a final concentration of 1 × 10^5^ conidia per mL and shaken at 28°C, 180 rpm for 6 days. Samples were removed at various times and centrifuged at 2800 ×g for 10 minutes. The supernatant was analyzed for extracellular enzyme activity. Experiments were carried out in triplicate and twice.

### 2.3. Enzymatic Assays

Total cellulase activity assay was analyzed on filter paper (filter paper activity—FPA), according to Ghose [23]. *β*-glycosidase activity was measured using salicin as the substrate, according to Chahal [24]. Endoglucanase activity was determined according to Ghose [23] using 2% (w/v) carboxymethylcellulose solution in citrate buffer, pH 4.8. Xylanase activity was determined in the same way as endoglucanase activity, except that 1% xylan from oat spelt solution was used as the substrate in place of carboxymethylcellulose. The concentration of reducing sugar was estimated as either xylose or glucose equivalents by the dinitrosalicylic acid (DNS) method according to Miller [25].

One unit (U) of enzyme activity was defined as the amount of enzyme required to liberate 1 *μ*mol of reducing sugar from the appropriate substrate per minute per mL of crude filtrate under the assay conditions.

### 2.4. Analytical Methods

The mycelial biomass was estimated by the quantity of N-acetylglucosamine according to the method described in Reissig et al. [26]. The Klason lignin of the samples was determined by the method described by Templeton and Ehrman (1995) [27].

### 2.5. Statistical Tests

The results were statistically analyzed using the PrismGraphPad program (GraphPad Software, Inc., CA, USA) to perform analysis of variance with Tukey's post hoc test for a *P* < 0.05.

## 3. Results and Discussion

Different pretreatments of sugar cane bagasse were tested to determine which were optimal for using bagasse as a component of the medium for cellulase production by* P. echinulatum*. The efficiency of the pretreatments was measured by determining Klason lignin (Table 1). It was found that the use of greater reagent concentrations in the pretreatments resulted in higher delignification indices, measured as the amount of lignin present in the substrate after the different pretreatments.

According to Kim and Holtzapple (2006) [28], an effective lignocellulose treatment process should remove all of the acetyl groups and reduce the lignin content to about 10% in the treated biomass; this reduction was obtained in the samples of this work. Additionally, Kong et al. (1992) [29] reported that alkalis remove acetyl groups from hemicellulose, thereby reducing the steric hindrance of hydrolytic enzymes and greatly enhancing carbohydrate digestibility. This reduction of crystallinity may be because H_2_O_2_ pretreatment can swell and dissolve cellulose, while NaOH can even penetrate into the amorphous area of cellulose and destruct the neighboring crystalline regions [30] (Wang et al. 2008). EDTA is used in the process to prevent the decomposition of peroxide [31]. The anthraquinone also has important function during the pretreatment, since it is agent which reduces lignin intermediates formed during pulping and thereby prevents the lignin intermediates from condensing during pulping [32].

Enzyme production experiments were carried out using samples of sugar cane bagasse from different treatments to verify secretion of cellulases and xylanases by* P. echinulatum*, strain 9A02S1. As controls, cultures were grown with microcrystalline cellulose and untreated sugar cane bagasse (Tables 2, 3, 4, and 5).

Table 2 shows that most cultures with pretreated bagasse had activity levels greater than or similar to culture T15 (cellulose only) and culture T16 (untreated bagasse). Comparing the performance of the cultures grown with sugar cane bagasse pretreated with high concentration (6 : 1) or low concentration (3 : 1) of reagents, on the sixth day of cultivation the activities of cultures T6 (1.232 ± 0.128 U·mL^−1^) and T12 (1.130 ± 0.098 U·mL^−1^)—for which the sugar cane bagasse was treated with half of the reagent concentrations—were similar to those of culture T5 (1.253 ± 0.147 U·mL^−1^) whose bagasse was treated with the double of the concentration of reagents.

Decreases in FPA values observed in cultures T11 and T12 on the fourth day could not be explained; however, it is possible that protease activity was present during this period, since previous observations showed that the secretion of proteases into the medium might reduce enzymatic activity [33].

Induction of endoglucanase activity was noted in cultures with sugar cane bagasse; however, it was higher in media where the sugar cane bagasse had been pretreated (Table 3). Table 2 shows that most cultures with pretreated bagasse activities higher than or similar to culture T15 (control with only cellulose) and higher activity than culture T16 (untreated sugar cane bagasse). These data show that pretreated bagasse can be used to induce the production of endoglucanases. The data also suggest that for the purpose of producing endoglucanases, cultivation may be interrupted on the fourth day, since afterwards only small changes in enzymatic activity and consequently a decrease in productivity are observed.

In most treatments, the *β*-glucosidase activity is higher after longer periods of culture (Table 4). On the fifth day, a peak in the *β*-glucosidase enzymatic activity was observed in culture T10, the culture with the highest enzymatic activity (0.288 ± 0.057 U·mL^−1^), while on the sixth day of cultivation cultures T4, T5, T6, T7, T12, T13, T14, and T16 showed values similar to those obtained from the control culture, T15. However the use of pretreated conditions is not economically attractive in terms of *β*-glucosidase production but is important for the other enzymes for lignocellulosic degradation. In previous works with enzymes of* P. echinulatum* [18], high relationships of FPA/*β*-glucosidase were obtained; these differences can be attributed to the different inducer substrate or the different substrate used to *β*-glucosidase determination. In some cases salicin is used and in others 4-nitrophenyl *β*-D-glucopyranoside.

Concerning xylanase activity, Table 5 shows that the cultures grown with cellulose (T15) or untreated sugar cane bagasse (T16) had higher enzymatic activities than or similar activities to cultures grown with pretreated sugar cane bagasse (6 : 1 and 3 : 1) throughout the duration of the experiment.

The lower xylanase activities observed in the cultures grown with pretreated sugar cane bagasse were probably due to the reduction of the inducing substrate (xylan) during the bagasse pretreatment. However, there cannot be a requirement for the presence of xylan for the induction of xylanases because the culture grown with cellulose alone also showed xylanase activity. It is well documented that the carbon source is an important variable in xylanase production, with lignocellulosic materials seeming to be better substrates than xylan for the production of xylanolytic enzymes [34–37]. Conversely,* T. reesei* showed the highest levels of endoxylanase activity when grown on cellulose [37]. This effect is probably due to the fact that the cellulase regulator ACEII also influences xylanase regulation. Therefore, the presence of cellulose may induce not only the cellulase production, but also the production of xylanase [38]. However, the experiments performed by Aro et al. [38] were done with crystalline cellulose and it is possible that amorphous cellulose from the alkali pretreatment have not the same inducing potential than the crystalline cellulose.

Although the enzyme activities obtained in this work are not high, or in a few times higher than those observed for other fungi, values that are compatible are found (Table 6).

**Figure 1:**
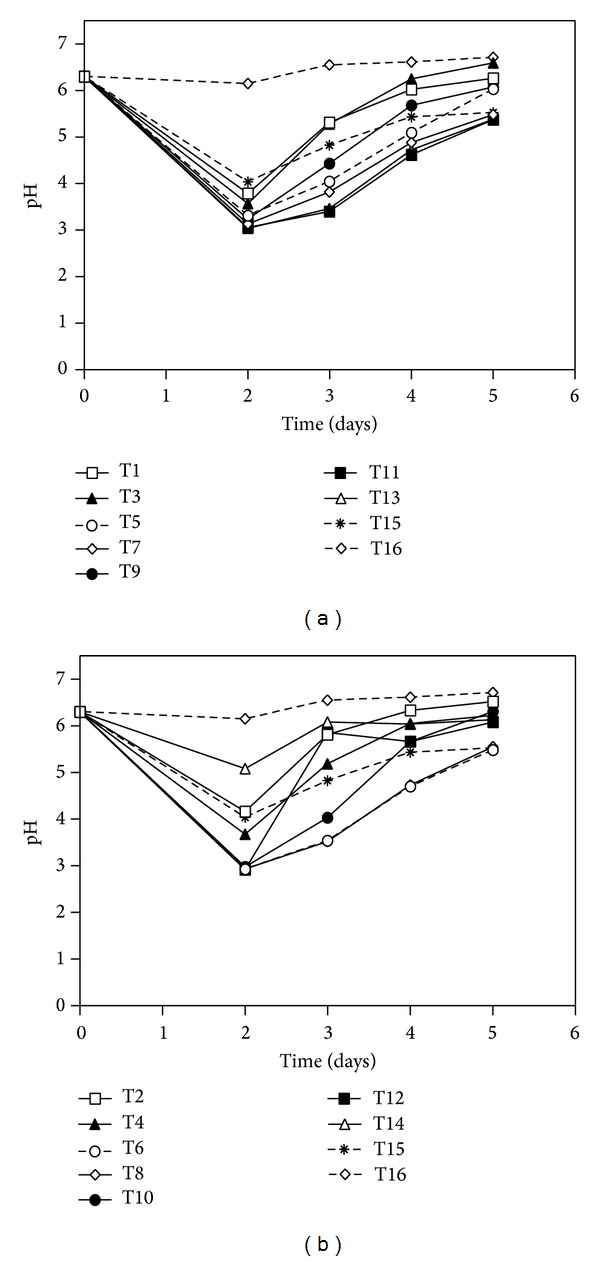
The pH variation of* Penicillium echinulatum* strain 9A02S1 submerged cultures in medium containing 1% w/v bagasse samples or cellulose.  Figure 1(a) shows the results for cultures grown with sugar cane bagasse pretreated with 6 parts of solution to 1 part of bagasse (6 : 1), and Figure 1(b) shows the results for cultures grown with sugar cane bagasse pretreated with 3 parts of solution to 1 part of bagasse (3 : 1).

**Figure 2:**
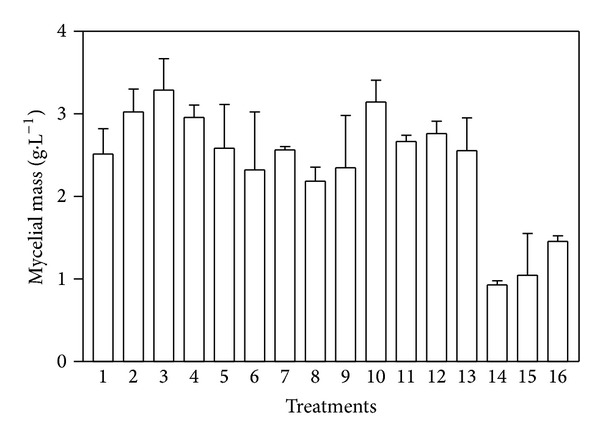
The quantity of mycelial mass produced by* Penicillium echinulatum* strain 9A02S1 in submerged culture in medium containing 1% w/v bagasse samples or cellulose on the sixth day of cultivation.

Figure 1 shows the pH variation during cultivation. A decrease in pH was noted in all cultures using both the 6 : 1 and 3 : 1 pretreated sugar cane bagasse and in the cellulose control culture (T15). The only culture not showing a decrease in pH was the one containing untreated sugar cane bagasse (T16). According to Sternberg and Dorval [37], the decrease in pH for* Trichoderma reesei* culture result of ammonia uptake during growth. The lack of growth in T16 can also be seen in Figure 2, which shows the quantity of mycelial mass estimated by amount of N-acetylglucosamine. With the exception of culture T14, cultures supplemented with pretreated sugar cane bagasse showed higher amounts of mycelial mass than those grown with cellulose (T15) and untreated sugar cane bagasse (T16) on the sixth day of cultivation.

On analyzing the enzymatic activities observed in this study, one can conclude that the production of FPA, endoglucanases, and *β*-glucosidases is favored in media supplemented with pretreated sugar cane bagasse relative to medium containing microcrystalline cellulose (T15). These results indicate the importance of pretreating sugar cane bagasse for use as a feedstock in the production of cellulases. Studies carried out with* Penicillium janthinellum, *strain NCIM 1171, have also shown that the xylanase and *β*-glucosidase activities in media supplemented with pretreated sugar cane bagasse were higher than the activities obtained in the medium supplemented with pure cellulose [5].

The differences observed for* P. echinulatum *and* P. janthinellum* relative to *β*-glucosidase and xylanase activities in media supplemented with pretreated sugar cane bagasse may be due to different concentrations of alkali employed in the pretreatment that results in different biomass composition and/or different mechanisms of enzyme production between mutants in response to the substrate provided [5].

In addition, the* Trichoderma viride* strain NCIM also showed higher xylanase activity in culture with pretreated bagasse than in culture with pure cellulose [2]. However, studies carried out with the* Trichoderma reesei* strain NRRL 3653 showed that a standard medium with pure cellulose resulted in higher growth rate and enzymatic activity after 144 h of cultivation compared to cultures supplemented with pretreated sugar cane bagasse, indicating that these microorganisms use pure cellulose more efficiently that the cellulose of sugar cane bagasse [39].

The lowest FPA, endoglucanase, and *β*-glucosidase activities were observed in cultures with untreated sugar cane bagasse. This sample has lignocellulose in its natural structure, thus preventing enzyme access to the substrate and limiting microbial growth [40].

The difficulty of cellulose degradation is consistent with the growth data provided in Figure 2, showing that the culture containing untreated sugar cane bagasse (T16) had a lower mycelial mass compared to the other cultures containing pretreated sugar cane bagasse, except for culture T14, in which enzymatic secretion and mycelial mass were lower, despite having one of the lowest levels of Klason lignin (4.577%). Kansoh et al. [40], studying cultures of* T. reesei* strain NRRL 3653, found lower levels of mycelial biomass in media containing untreated sugar cane bagasse compared to cultures with alkaline pretreated biomass.

Although the control culture with pure cellulose had lower quantities of mycelial biomass than the cultures with pretreated sugar cane bagasse, enzymatic activities were similar, perhaps indicating that pure cellulose is a stronger inducer of the enzymatic complex than sugar cane bagasse. Furthermore, the data indicate that the pretreated sugar cane bagasse was efficiently converted into mycelial biomass. These results suggest the possibility of growing cultures using sugar cane bagasse to form biomass in combination with another cellulase inducer to increase the production of cellulolytic enzymes.

The difference in the enzymatic activities of the cultures using pretreated sugar cane bagasse may be due the reduction of lignin in the biomass or to variation in the proportions of amorphic and crystalline cellulose in the samples after pretreatment. This potential variation in the quantity and structure of the cellulose in the samples of pretreated sugar cane bagasse may be attributed to the ability of each pretreatment to break the hydrogen bonds in the crystalline form of cellulose. Consequently, cultures with a greater availability of accessible cellulose may generate a greater quantity of mycelial biomass. Both the physical structure of sugar cane bagasse cellulose and the enzymatic activity of the complex used in its hydrolysis are important factors that influence the efficiency of the conversion of cellulose into other products.

Curiously, the lowest FPA, endoglucanase, and *β*-glucosidase activities were observed in the culture with untreated sugar cane bagasse, despite the fact that this culture showed the greatest quantities of xylanases. However, these results can be explained by the presence of available hemicellulose in the untreated sugar cane bagasse, besides the cellulose that is present in this sample. These data suggest that a combination of pretreated and untreated sugar cane bagasse may be optimal for cultures when the aim is to produce enzymatic complexes rich in FPA, endoglucanases, *β*-glucosidases, and xylanases. In addition, the results obtained indicate that the removal of lignin from the residues is not the only factor influencing the production of these enzymes, since there was no direct relationship between the quantity of lignin and the enzymatic activities.

## 4. Conclusions

The results of this study indicate the formation of* P. echinulatum* biomass in cultures grown with sugar cane bagasse as the carbon source, along with the production of FPA, endoglucanase, *β*-glucosidase, and xylanase activities in the enzymatic broth. These data suggest that cellulose may be partially or totally replaced by pretreated sugar cane bagasse for the production of FPA, endoglucanases, and *β*-glucosidases and by untreated sugar cane bagasse for the production of xylanases in cultures of* P. echinulatum *for the purpose of cellulase and xylanase production. The incorporation of cheap cellulose sources such as sugar cane bagasse into media for producing lignocellulose enzymes should contribute to a decrease in the production costs of enzymatic complexes capable of hydrolyzing lignocellulose residues to form fermented syrups, thus contributing to a cost-efficient production of bioethanol.

## Figures and Tables

**Table 1:** Lignin percentages obtained from different pretreatments using sugar cane bagasse.

Treatment	Solution used	Proportion of solution (w) : sugar cane bagasse (w)	Lignin (%)
T1	16% NaOH	6 : 1	9.95 ± 0.09
T2	16% NaOH	3 : 1	11.61 ± 0.08
T3	16% NaOH + 0.3% H_2_O_2 _	6 : 1	6.49 ± 0.13
T4	16% NaOH + 0.3% H_2_O_2 _	3 : 1	5.55 ± 0.12
T5	16% NaOH + 0.3% H_2_O_2_ + 0.02% AQ	6 : 1	9.24 ± 0.06
T6	16% NaOH + 0.3% H_2_O_2_ + 0.02% AQ	3 : 1	4.69 ± 0.14
T7	16% NaOH + 0.3% H_2_O_2_ + 0.02% AQ + 0.3% EDTA	6 : 1	7.16 ± 0.12
T8	16% NaOH + 0.3% H_2_O_2_ + 0.02% AQ + 0.3% EDTA	3 : 1	3.89 ± 0.19
T9	16% NaOH + 0.6% H_2_O_2 _	6 : 1	4.58 ± 0.21
T10	16% NaOH + 0.6% H_2_O_2 _	3 : 1	4.16 ± 0.19
T11	16% NaOH + 0.6% H_2_O_2_ + 0.02% AQ	6 : 1	5.41 ± 0.18
T12	16% NaOH + 0.6% H_2_O_2_ + 0.02% AQ	3 : 1	2.75 ± 0.12
T13	16% NaOH + 0.6% H_2_O_2_ + 0.02% AQ + 0.3% EDTA	6 : 1	2.78 ± 0.08
T14	16% NaOH + 0.6% H_2_O_2_ + 0.02% AQ + 0.3% EDTA	3 : 1	4.58 ± 0.19
T15	Cellulose E (control)	—	—
T16	Untreated bagasse (control)	—	22.46 ± 0.86

**Table 2:** The filter paper activity of *Penicillium echinulatum* strain 9A02S1 in submerged culture in medium containing 1% w/v bagasse samples or cellulose.

Treatment	Filter paper activity (U·mL^−1^) Time of process (days)			
	3	4	5	6
T1	0.448 ± 0.099^ab^	0.737 ± 0.102^ab^	0.784 ± 0.183^ab^	1.008 ± 0.142^ab^
T2	0.408 ± 0.138^ab^	0.687 ± 0.148^ab^	0.707 ± 0.073^bc^	0.573 ± 0.258^c^
T3	0.598 ± 0.036^a^	0.556 ± 0.136^b^	0.706 ± 0.166^bc^	0.960 ± 0.106^ab^
T4	0.478 ± 0.276^ab^	0.730 ± 0.205^ab^	0.770 ± 0.233^ab^	1.013 ± 0.129^ab^
T5	0.745 ± 0.041^a^	0.784 ± 0.272^ab^	1.150 ± 0.129^a^	1.253 ± 0.147^a^
T6	0.522 ± 0.019^a^	0.837 ± 0.072^ab^	1.014 ± 0.126^ab^	1.232 ± 0.128^a^
T7	0.705 ± 0.175^a^	0.755 ± 0.240^ab^	0.867 ± 0.264^ab^	1.093 ± 0.174^ab^
T8	0.513 ± 0.162^a^	0.749 ± 0.057^ab^	0.842 ± 0.077^ab^	0.965 ± 0.065^ab^
T9	0.608 ± 0.125^a^	0.954 ± 0.181^ab^	0.856 ± 0.010^ab^	0.965 ± 0.061^ab^
T10	0.519 ± 0.068^a^	0.722 ± 0.147^ab^	0.757 ± 0.053^ab^	0.847 ± 0.073^bc^
T11	0.715 ± 0.102^a^	0.125 ± 0.008^c^	1.127 ± 0.175^ab^	1.087 ± 0.118^ab^
T12	0.690 ± 0.263^a^	0.135 ± 0.001^c^	1.036 ± 0.135^ab^	1.130 ± 0.096^ab^
T13	0.750 ± 0.036^a^	0.987 ± 0.083^a^	1.067 ± 0.104^ab^	1.014 ± 0.117^ab^
T14	0.071 ± 0.022^b^	0.127 ± 0.002^c^	0.000 ± 0.000^d^	0.114 ± 0.028^d^
T15	0.624 ± 0.084^a^	0.646 ± 0.144^ab^	0.945 ± 0.099^ab^	0.973 ± 0.065^ab^
T16	0.503 ± 0.187^a^	0.424 ± 0.062^bc^	0.305 ± 0.111^cd^	0.524 ± 0.013^c^

Values (means) with the same letters for the same day did not differ significantly by Tukey's test (*P* < 0.05).

**Table 3:** The endoglucanase activities of *Penicillium echinulatum* strain 9A02S1 in submerged culture in medium containing 1% w/v bagasse samples or cellulose.

Treatment	Endoglucanases (U·mL^−1^) Time of process (days)			
	3	4	5	6
T1	0.456 ± 0.076^ab^	1.028 ± 0.307^ab^	1.114 ± 0.158^ab^	1.442 ± 0.064^a^
T2	0.405 ± 0.144^ab^	1.376 ± 0.401^a^	0.950 ± 0.189^ab^	1.408 ± 0.089^a^
T3	0.383 ± 0.060^ab^	1.507 ± 0.118^a^	1.372 ± 0.343^ab^	1.573 ± 0.233^a^
T4	0.298 ± 0.048^bc^	1.282 ± 0.597^a^	1.603 ± 0.015^a^	1.590 ± 0.130^a^
T5	0.720 ± 0.083^a^	1.079 ± 0.183^ab^	1.583 ± 0.299^a^	1.795 ± 0.185^a^
T6	0.619 ± 0.216^ab^	1.552 ± 0.460^a^	1.243 ± 0.279^ab^	1.676 ± 0.179^a^
T7	0.240 ± 0.062^c^	0.239 ± 0.058^b^	1.350 ± 0.096^ab^	1.630 ± 0.067^a^
T8	0.161 ± 0.030^c^	1.395 ± 0.340^a^	1.179 ± 0.355^ab^	1.410 ± 0.035^a^
T9	0.418 ± 0.072^ab^	1.151 ± 0.187^ab^	1.221 ± 0.303^ab^	1.521 ± 0.078^a^
T10	0.408 ± 0.117^ab^	1.528 ± 0.495^a^	1.239 ± 0.123^ab^	1.465 ± 0.088^a^
T11	0.697 ± 0.061^a^	1.580 ± 0.306^a^	1.541 ± 0.176^a^	1.623 ± 0.112^a^
T12	0.443 ± 0.135^ab^	1.318 ± 0.000^a^	1.202 ± 0.028^ab^	1.614 ± 0.146^a^
T13	0.286 ± 0.006^bc^	1.231 ± 0.265^a^	1.557 ± 0.063^a^	1.846 ± 0.454^a^
T14	0.274 ± 0.026^bc^	1.231 ± 0.265^a^	1.557 ± 0.063^a^	1.846 ± 0.454^a^
T15	0.416 ± 0.089^abc^	1.105 ± 0.269^ab^	1.584 ± 0.449^a^	1.602 ± 0.087^a^
T16	0.457 ± 0.213^abc^	0.785 ± 0.029^b^	0.832 ± 0.214^b^	1.246 ± 0.114^a^

Values (means) with the same letters for the same day did not differ significantly by Tukey's test (*P* < 0.05).

**Table 4:** The *β*-glucosidase activities of *Penicillium echinulatum* strain 9A02S1 in submerged culture in medium containing 1% w/v bagasse samples or cellulose.

Treatment	*β*-glucosidases (U·mL^−1^) Time of process (days)			
	3	4	5	6
T1	0.018 ± 0.000^b^	0.099 ± 0.005^a^	0.176 ± 0.023^b^	0.143 ± 0.019^b^
T2	0.018 ± 0.000^b^	0.092 ± 0.002^a^	0.160 ± 0.017^b^	0.112 ± 0.032^bc^
T3	0.018 ± 0.000^b^	0.092 ± 0.001^a^	0.135 ± 0.017^bc^	0.005 ± 0.005^c^
T4	0.018 ± 0.000^b^	0.093 ± 0.001^a^	0.135 ± 0.000^bc^	0.187 ± 0.057^ab^
T5	0.018 ± 0.000^b^	0.092 ± 0.001^a^	0.068 ± 0.014^cd^	0.162 ± 0.043^ab^
T6	0.018 ± 0.000^b^	0.091 ± 0.000^a^	0.167 ± 0.009^b^	0.182 ± 0.014^ab^
T7	0.018 ± 0.000^b^	0.092 ± 0.001^a^	0.153 ± 0.016^bc^	0.154 ± 0.030^ab^
T8	0.018 ± 0.000^b^	0.091 ± 0.001^a^	0.152 ± 0.021^bc^	0.026 ± 0.008^c^
T9	0.018 ± 0.000^b^	0.091 ± 0.000^a^	0.141 ± 0.011^bc^	0.017 ± 0.008^c^
T10	0.018 ± 0.000^b^	0.093 ± 0.001^a^	0.288 ± 0.058^a^	0.155 ± 0.053^b^
T11	0.017 ± 0.001^b^	0.063 ± 0.047^a^	0.126 ± 0.022^bc^	0.040 ± 0.013^c^
T12	0.018 ± 0.006^b^	0.091 ± 0.002^a^	0.153 ± 0.097^bc^	0.221 ± 0.048^ab^
T13	0.012 ± 0.000^c^	0.086 ± 0.009^a^	0.142 ± 0.016^bc^	0.243 ± 0.006^a^
T14	0.000 ± 0.000^d^	0.091 ± 0.001^a^	0.021 ± 0.000^d^	0.188 ± 0.016^ab^
T15	0.029 ± 0.000^a^	0.092 ± 0.002^a^	0.131 ± 0.024^bc^	0.215 ± 0.012^ab^
T16	0.018 ± 0.001^b^	0.091 ± 0.001^a^	0.082 ± 0.008^cd^	0.196 ± 0.001^ab^

Values (averages) with the same letters for the same day do not differ significantly by Tukey's test (*P* < 0.05).

**Table 5:** The xylanase activities of *Penicillium echinulatum* strain 9A02S1 in submerged culture in medium containing 1% w/v bagasse samples or cellulose.

Treatment	Xylanases (U·mL^−1^) Time of process (days)			
	3	4	5	6
T1	0.871 ± 0.079^b^	0.710 ± 0.085^bc^	0.638 ± 0.132^b^	0.338 ± 0.007^c^
T2	0.984 ± 0.083^b^	0.894 ± 0.031^b^	0.813 ± 0.028^b^	0.376 ± 0.005^abc^
T3	0.871 ± 0.023^b^	0.612 ± 0.095^c^	0.568 ± 0.039^bc^	0.294 ± 0.028^c^
T4	0.918 ± 0.270^b^	0.280 ± 0.015^d^	0.734 ± 0.207^b^	0.389 ± 0.026^abc^
T5	0.951 ± 0.127^b^	0.733 ± 0.052^bc^	0.774 ± 0.122^b^	0.398 ± 0.055^abc^
T6	0.966 ± 0.084^b^	0.874 ± 0.106^b^	0.830 ± 0.080^b^	0.440 ± 0.026^ab^
T7	0.912 ± 0.062^b^	0.638 ± 0.062^c^	0.677 ± 0.084^b^	0.367 ± 0.035^bc^
T8	0.877 ± 0.043^b^	0.730 ± 0.077^bc^	0.673 ± 0.195^b^	0.442 ± 0.066^ab^
T9	0.905 ± 0.052^b^	0.535 ± 0.075^cd^	0.577 ± 0.004^bc^	0.326 ± 0.025^c^
T10	0.962 ± 0.093^b^	0.565 ± 0.032^cd^	0.554 ± 0.143^bc^	0.291 ± 0.104^c^
T11	0.932 ± 0.068^b^	0.302 ± 0.081^d^	0.805 ± 0.054^b^	0.362 ± 0.054^bc^
T12	0.701 ± 0.317^b^	0.489 ± 0.318^cd^	0.745 ± 0.375^bc^	0.430 ± 0.011^abc^
T13	0.879 ± 0.106^b^	0.704 ± 0.029^bc^	0.729 ± 0.039^b^	0.400 ± 0.036^abc^
T14	0.190 ± 0.031^c^	0.270 ± 0.069^d^	0.090 ± 0.000^d^	0.000 ± 0.000^d^
T15	1.486 ± 0.082^a^	1.022 ± 0.143^ab^	1.046 ± 0.154^ab^	0.489 ± 0.071^ab^
T16	1.457 ± 0.273^a^	1.212 ± 0.072^a^	1.179 ± 0.048^a^	0.517 ± 0.070^a^

Values (means) with the same letters for the same day did not differ significantly by Tukey's test (*P* < 0.05).

**Table 6:** Comparison of cellulase production by fungi in sugar cane bagasse pretreated by different methods.

Microorganism	Substrate and concentration (%, w/v)	Enzyme activity (U·mL^−1^)			Reference
		FPA	Endoglucanase	*β*-glucosidase	
*P. echinulatum *9A02S1	Alkaline pretreated sugar cane bagasse	1.13	1.76	0.21	This work
*P. echinulatum *9A02S1	Biological pretreated sugar cane bagasse	0.13	1.0	0.18	[3]
*P. funiculosum* ATCC 11797	Glucose (0.4%) and sugar cane bagasse (1.5%) after acid and alkaline pretreatment	1.13	10.25	2.26	[20]
*T. harzianum* IOC 3844	Cellulignin from sugar cane bagasse by using two consecutive pretreatments (acid and alkaline)	0.001	0.027	0.609	[21]
*T. harzianum* L04	Partially delignified cellulignin	0.04	0.012	0.01	[22]
